# Performance Comparison of Centered and Tilted Blunt and Lighthouse Tip Cannulae for Drainage in Extracorporeal Life Support

**DOI:** 10.1007/s13239-024-00770-x

**Published:** 2025-02-10

**Authors:** Federico Rorro, Lars Mikael Broman, Lisa Prahl Wittberg

**Affiliations:** 1 FLOW, Department of Engineering Mechanics, KTH Royal Institute of Technology, Stockholm, Sweden; 2 ECMO Centre Karolinska, Pediatric Perioperative Medicine and Intensive Care, Karolinska University Hospital, Stockholm, Sweden; 3 Department of Physiology and Pharmacology, Karolinska Institutet, Stockholm, Sweden

**Keywords:** Extracorporeal membrane oxygenation (ECMO), Particle image velocimetry (PIV), Cannula, Drainage

## Abstract

**Introduction:**

Extracorporeal membrane oxygenation is a lifesaving treatment for patients with refractory acute respiratory, circulatory, or combined cardiopulmonary failure. The patient is cannulated with one or two cannulae for drainage and reinfusion of blood. Blood is drained from the patient, pumped through a membrane lung for oxygenation and returned to the patient. Treatment efficiency depends on correct cannula positioning and interactions between drainage and reinfusion cannula.

**Methods:**

An experimental setup was built to study the isolated drainage performance of 24 Fr rigid models of a blunt and lighthouse tip cannula, both when centered and when tilted towards the vessel wall. Planar particle image velocimetry was used to investigate the flow field with water as the fluid medium.

**Results:**

For similar flow configuration, higher shear stresses were recorded in the blunt tip rather than lighthouse tip cannula. Moreover, in the lighthouse tip cannula, side-holes furthest from the tip (*proximal* side-holes) had the highest drainage. Results did not change substantially when the cannula was tilted towards the vessel wall.

**Conclusions:**

The effective drainage point of the lighthouse tip cannula was located near the proximal side-holes. Lower shear stresses were recorded in the lighthouse tip cannula when compared with the blunt tip cannula, for all considered flow rate ratios and cannula positions.

**Supplementary Information:**

The online version contains supplementary material available at 10.1007/s13239-024-00770-x.

## Introduction

Extracorporeal membrane oxygenation (ECMO) is a lifesaving treatment for patients with refractory acute respiratory, circulatory, or combined cardiopulmonary failure first used successfully in 1972 by Hill et al. [1]. Before the publication of the CESAR trial in 2009 [2], ECMO treatment of adults was limited. Since then, the endorsement of ECMO by centers around the world for patients with refractory acute cardio/pulmonary failure has increased, encompassing more than 570 certified centers in 2023 [3].

The ECMO circuit has a centrifugal or roller pump to drive the flow, a membrane lung (oxygenator) for oxygenation and carbon dioxide removal, and tubing with connectors. To provide treatment, vascular accesses for blood drainage and return are obtained via patient cannulation. Depending on the patient’s need, veno-venous (VV) or veno-arterial (VA) ECMO can be initiated. VV ECMO offers respiratory support with both drainage and return on the venous side, generally involving jugular and/or femoral veins using two single lumen or one dual-lumen cannula. VA ECMO provides both circulatory and respiratory support. A typical configuration uses drainage from the inferior vena cava (IVC) with return via a femoral artery [4]. Regardless of the type of support offered, the aim is to drain blood with the lowest oxygen content from the venous side of the patient’s circulation and return the oxygenated blood at a site for optimal oxygen delivery. Although lifesaving, the interaction between technology and patient may introduce complications related directly to mechanical properties (e.g. shear forces on blood leading to hemolysis, bleeding, and thrombus formation) [5, 6]. Further, ECMO is associated with complications such as limb ischemia, neurological injury, or neurocognitive changes besides technical problems, e.g. failure of the blood pump, oxygenator, and cannulae [7–9].

For these reasons, the characterization of the performance of cannulae and other circuit components is crucial to understand blood flow dynamics within the isolated artificial components as well as in combination with the native circulation. For cannulae in drainage configuration, side-holes provide multiple (“adaptive”) drainage points in case of hole occlusion, although with penalty of slight reduction in drainage flow. Further, through computational fluid dynamics (CFD), lower shear stress levels were reported in cannulae with side-holes [10]. Goto et al. [11] showed that increasing the number of side-holes (while maintaining the total orifice area constant) increased shear stress levels and decreased drainage flow. Slanted side-holes, compared to side-holes perpendicular to the cannula body, showed reduction in shear rate and reduced flow loss [12]. Further, a recent study by Rauh et al. [13] compared numerical simulations and magnetic resonance imaging of the flow in a lighthouse (single-stage) tip cannula. It was reported that the side-holes furthest from the tip (proximal side-holes) were characterized by having the highest drainage fraction. Moreover, when entering through the cannula side-holes, the flow was described to form a Y-shaped inflow. Numerically modifying the fluid model from Newtonian to non-Newtonian did not alter the drainage performance of the cannula or the flow behavior in the side-holes region of a lighthouse tip cannula [14].

Experimentally, studies have been performed on different cannula models, comparing the various designs based on the relation between recorded flow rate and pressure drop across the cannula during drainage [15]. During pulsatile support, no significant difference was found in critical blood parameters when comparing the performance of five different cannula tip designs [16]. In particular, minimal differences of velocity in the IVC were found when pulsatile or steady IVC inflow conditions were numerically compared [17]. A recent study by Vatani et al. experimentally and numerically analyzed the lighthouse tip cannula drainage performance with various distances between side-holes [18]. Vatani et al. confirmed the results of highest drainage at the proximal side-holes and the Y-shaped structure inside the cannula body reported by Rauh et al. [13]. The numerical study by Fiusco et al. [14] related the Y-shaped structure to the pressure difference across the cannula walls, with higher pressure outside the cannula (due to lower velocity magnitudes) when compared to the pressure inside the cannula (due to higher velocity magnitudes). The pressure difference had the highest value close to the proximal side-holes and the magnitude reduced when moving towards the tip.

Mixing performance of two cannula designs were experimentally investigated in return configuration by Lemétayer et al. [19], showing similar mixing length but reduced shear stresses and recirculation on the side of a non-centered cannula. Further, the effect on flow structures and shear stresses of a non-centered return cannula at various distances from the wall was numerically investigated, showing increased wall shear stress levels and mixing with a reduction of distance between the cannula tip and the wall [20].

Most of the aforementioned studies, with few exceptions [19, 20], only considered a cannula centered within the vessel. Besides these studies, there is a limited number of experimental studies performed on cannulae for drainage or return with either a centered or tilted cannula. Further, no investigation has focused on the drainage fraction through each cannula orifice for different cannula positions within the containing vessel or with different flow rate of cannula and outer flow. This study experimentally investigated the flow characteristics of a rigid blunt and a rigid lighthouse tip cannula in drainage configuration. For the first time, drainage fraction across all cannula orifices was estimated for both centered and laterally shifted cannula. Moreover, different cannula to outer flow rate ratios were investigated to evaluate the impact on drainage performance as well as the flow and shear stress characteristics in the two cannula designs. Data obtained in this study, and available in the supplemental material, can be used for validation of numerical studies.

## Experimental Methodology and Case Setup

### Experimental Setup

**Fig. 1:**
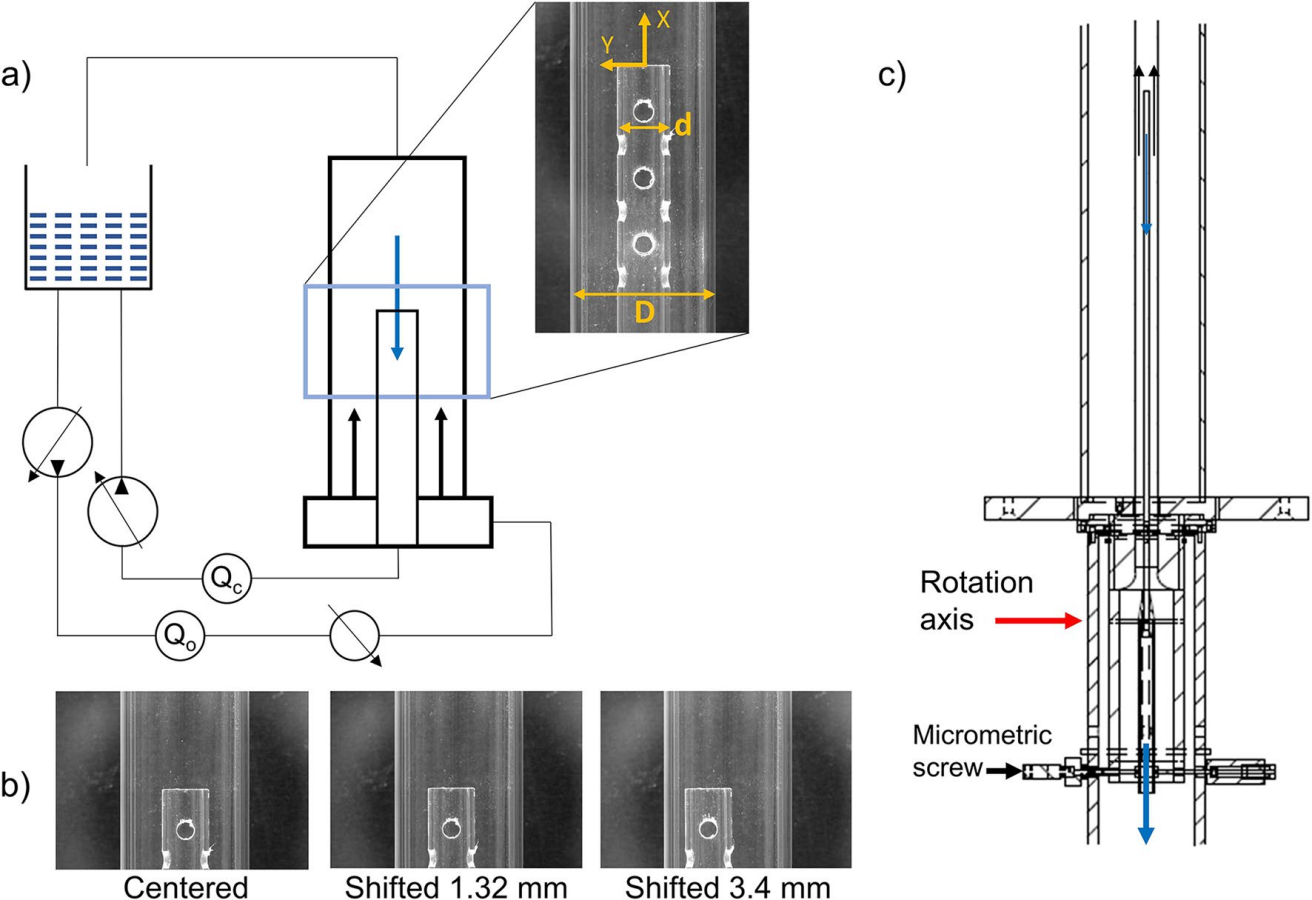
Setup overview with **a** experimental loop schematics, **b** the different cannula positions investigated ($$r_c=0, 1.32, 3.4$$ mm), **c** detailed view of the system used to shift the cannula. Flow direction is marked with blue arrows for the cannula flow ($$Q_c$$) and black arrows for the co-flow ($$Q_o)$$.

The experimental setup, similar to the one used by Lemétayer et al. [19], consisted of two coaxial glass tubes placed in a rectangular box filled with water to reduce optical aberrations due to the circular shape of the tubes (Fig. 1). The outer tube had an inner diameter (D) of 18.3 mm, with dimension chosen according to values reported in literature concerning the IVC mean diameter [21, 22]. The IVC is a typical drainage point during VV or VA ECMO [4].

Two cannulae were studied, both corresponding to a 24 Fr^1^ cannula ($d = {8.0}$ mm) with inner diameters of $d_b = {5.6}$ mm for the blunt tip, and $d_{lh} = {6.0}$ mm for the lighthouse (single-stage) tip cannula. The coordinate system used had the origin in the center of the cannula tip, the *x*-axis oriented in the axial direction with positive values moving away from the cannula tip, while the *y*-axis was oriented in the radial direction with positive values towards the outer wall (Fig. 1a). The notations applied for the mean velocity, instantaneous velocities and root mean square (rms) fluctuations along the *x*-axis are *U*, *u*, and *u’* respectively. While on the *y*-axis the properties are reported as *V*, *v*, and *v’* respectively.

**Fig. 2:**
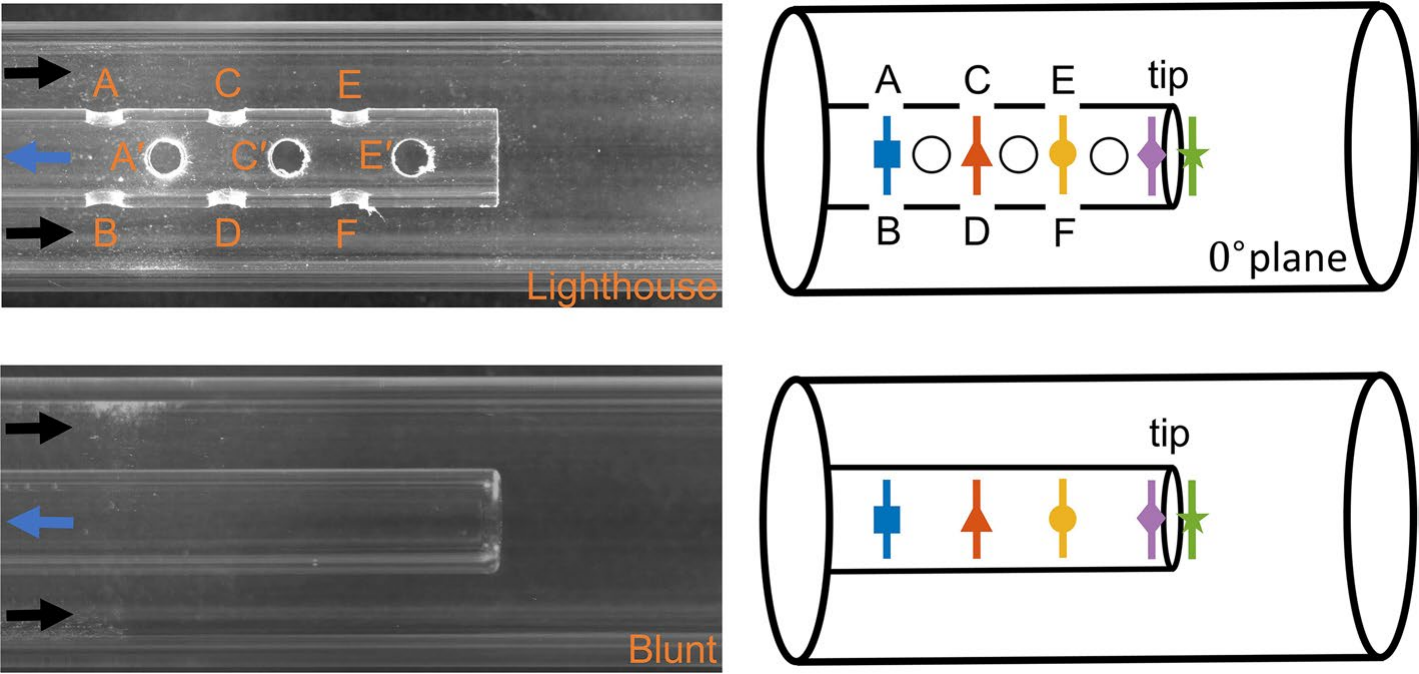
On the left, lighthouse and blunt tip cannula in the centered position and with side-holes marked with the used convention. On the right, schematic representation of cannula designs with definition of side-holes and position  of extracted velocity profiles on the $${0}^\circ$$ plane. Filled symbols are used for $${0}^\circ$$ plane while empty symbols are used for the $${90}^\circ$$ plane. Flow direction is marked with blue arrows for the cannula flow ($$Q_c$$) and black arrows for the co-flow ($$Q_o)$$

The blunt tip cannula had one single end-hole while the considered lighthouse tip cannula had one end-hole plus 12 side-holes distributed within 33.5 mm from the cannula tip (Fig. 2). The lighthouse tip cannula had side-holes arranged in four lines of three side-holes each, with 10 mm distance between centers of side-holes on the same line. Side-holes were arranged in a staggered configuration with two lines of side-holes starting at $x = - 12$ mm while the other two lines started at $x = - 7$ mm. As the proximal side-holes were included, the two lines starting at $x = - 12$ mm were studied first and are named with non-primed letters in the results (e.g. A, B, C, D, E, and F, see Fig. 2 for reference). This group of side-holes will from now on be referred to as the plane with angle of $0^\circ$. After moving the optical setup of lenses, mirror and camera data was also acquired on the cross plane with side-holes starting at $x = - 7$ mm. Side-holes studied in this configuration are marked with primed letters (e.g. A’, B’, C’, D’, E’, and F’) and referred as belonging to the plane with angle of ${90}^\circ$. The sequential acquisition of two cross planes through a single camera enabled the collection of data while preserving the same relative side-holes obstruction on the two planes when the cannula was shifted inside the containing vessel.

Figure 2 displays the two cannulae investigated in this study. On the left side of Fig. 2 pictures of the blunt (bottom) and lighthouse tip cannula (top) are displayed including positions and definition of all the visible side-holes on the ${0}^\circ$ plane for the lighthouse tip cannula. A schematic representation of the cannulae is visible to the right of Fig. 2 along with lines marking the location where data was extracted to compare flow velocity inside the cannulae. Velocity data was extracted along lines crossing the center of the side-holes belonging to the same row (i.e. opposing side-holes, marked with blue, red, and yellow lines) and also symmetrically with respect to the cannula tip (purple and green lines). Data extracted for the ${0}^\circ$ plane is reported with filled symbols while empty symbols are used for data for the ${90}^\circ$ plane.

As shown in Fig. 1, the experimental setup consisted of a common tank and two flow circuits each driven by a separate pump, the outer one for the co-flow representing the native venous flow ($Q_o$) and the inner controlling the cannula flow ($Q_c$). Steady flow was used in both cannula and outer vessel to isolate the effect of the cannula position and flow rate ratio on the drainage performance of the cannula. The tip of the 500 mm long cannula was placed at half height of the outer tube. The lengths of the tubes for the cannula and the co-flow were selected to ensure fully developed flow within each tube upstream of the cannula tip. To simulate conditions where the cannula tip is shifted towards one side of the outer vessel’s wall, the rigid glass cannula was shifted by 1.32 and 3.4 mm. The mounting system of the cannula had two fixed pins on both sides of the cannula defining a rotational axis, as marked in Fig. 1c. The displacement, controlled through a micrometer head, was measured between the centerline of the outer tube and the centerline of the cannula. The maximum displacement was limited by the dimensions of the outer tube, and the tilt angle ($\alpha$) never exceeded ${0.5}^\circ$. In the following, the displacement of the cannula is referred to as *lateral shift* with its tip position defined by $r_c$, similarly to Lemétayer et al. [19].

Water was used as fluid medium with a temperature of $20.5 \pm 0.5\,^\circ \hbox {C}$, due to fluid transparency and density value close to that of blood at ${37}\,^\circ \hbox {C}$. Use of a Newtonian fluid such as water was motivated by the need to control the fluid physical properties and due to the expected high level of shear rate [14]. For shear rates higher that $100\,\textrm{s}^{-1}$, blood behavior is assumed to be Newtonian with a viscosity 2 to 4 times that of water, depending on hematocrit (range of 20–40% based on ECMO center preference, [23, 24]). However, in low shear regions, the assumption of Newtonian fluid can induce uncertainties due to low shear rates that consequently lead to variability in viscosity.

### Case Setup

For the blunt tip cannula, twelve different cases were studied. While, for the lighthouse tip cannula, twelve cases were investigated for each cannula orientation leading to a total of twenty-four cases collected (twelve cases for ${0}^\circ$ plane and twelve cases for ${90}^\circ$ plane). The experiments were performed changing $Q_c$ as well as $Q_o$. Each cannula was studied when centered ($r_c ={0}$ mm) as well as laterally shifted ($r_c$ of 1.32 or 3.4 mm). When the cannula was shifted, the two cannula sides on the ${0}^\circ$ plane will be referred to as: *narrow side* for the side closest to the wall and *wide side* for the side furthest from the wall. As the cannula shift happened on the ${0}^\circ$ plane, the ${90}^\circ$ cases always had the cannula centered in the outer vessel. However, to ease comparison between the two planes, data acquired on the ${90}^\circ$ plane were reported with the shift of the cannula on the ${0}^\circ$ plane. To highlight the flow structures developing in the proximal side-hole region of the lighthouse tip cannula, close-up acquisitions were performed with a centered cannula (on the ${0}^\circ$ plane) for all considered flow configurations.

**Table 1:** Combinations of cannula position ($r_c$), cannula drainage flow ($Q_c$), co-flow ($Q_o$), and flow rate ratio *Q* examined

$r_c$ (mm)	$Q_c$ (L/min)	$Q_o$ (L/min)	$Q = Q_c/Q_o$ (–)
0/1.32/3.4	1.3	1.3	1
0/1.32/3.4	1.3	2.6	0.5
0/1.32/3.4	2.6	1.3	2
0/1.32/3.4	2.6	2.6	1

Flow rates were scaled using the Reynolds number to obtain dynamic similarity with conditions present in the IVC. Table 1 shows the cases investigated with the flow rates studied being combinations of 1.3 or 2.6 L/min. For co-flow ($Q_o$) of $Q_o = 1.3$ L/min the cannula drainage flow rates investigated were $Q_c=1.3$ L/min and $Q_c =2.6$ L/min leading to flow rate ratios ($Q=Q_c/Q_o$) of 1 and 2, respectively. For $Q_o=2.6$ L/min, cannula flow rates were set to $Q_c=2.6$ and 1.3 L/min resulting in flow rate ratios *Q* of 1 and 0.5, respectively. Each of the previous flow rate combination was studied both with the cannula centered ($r_c =0$ mm) as well as laterally shifted ($r_c$ of 1.32 or 3.4 mm).

### Measurement Technique

Two flow meters (VMZ081; SIKA, Kaufungen, Germany) were used, one positioned after the pump driving the co-flow and one between the drainage cannula and the drainage pump. The coordinate system used had the origin at the center of the cannula tip with the *x*-axis oriented in the axial direction while the *y*-axis is in the radial direction (Fig. 1a). The experiments were performed using planar particle image velocimetry (PIV). The interrogation window applied for data acquisition was located in a vertical plane crossing the cannula axis from 68 mm downstream to 35 mm upstream the cannula tip.

Titanium dioxide ($\text {TiO}_2$) particles with diameter of 5 $\upmu$m were used as seeding. The PIV system had a high speed Nd:YAG laser (LPY703 50-200, Litron Laser, Rugby, England) operating at 532 nm and one Dantec high-speed double frame camera (Speedsense M120, Dantec Dynamics, Skovlunde, Denmark). Acquisition for blunt and lighthouse tip cannula on the ${0}^\circ$ plane was performed using a 105 mm lens ($f/d_{max} = 1{:}1.8$ (Nikkor, Nikon, Tokyo, Japan)) mounted over a 20 mm extension ring. The close-up images for this configuration, to better resolve fluid structure within the lighthouse tip cannula in the proximal side-hole region, were acquired with the same camera while using a 50 mm lens ($f/d_{max} = 1{:}2.8$ (SIGMA, Kanagawa, Japan)) and no extension ring. Due to space constraints, data on the ${90}^\circ$ plane for the lighthouse tip cannula was also acquired with the 50 mm $f/d_{max} = 1{:}2.8$ SIGMA lens. The final magnification factors for the blunt and lighthouse tip cannulae at the ${0}^\circ$ plane was 27.5 and 56.7 pixel/mm for the 105 and 50 mm lens, respectively. Instead, data collected for the lighthouse tip cannula on the ${90}^\circ$ had a final magnification factor of 33.75 pixel/mm. The laser sheet was formed using a combination of mirrors and lenses and aligned to the center of the outer tube. The laser sheet thickness was 1 mm. Each image series acquired at 200 Hz contained 1339–1540 image pairs.

To recover the velocity field, the adaptive PIV algorithm within DynamicStudio v.2015a (Dantec Dynamics) was used. Each image pair of the series was pre-processed by removing the mean of the complete series to eliminate the cannula profile from the frame. The interrogation area (IA) went from $32\times 32$ pixels down to $24\times 24$ pixels. The percentage of spurious vectors never reached more than 1% for any of the presented cases. Due to laser reflection and diffraction on the outer tube and the cannula walls, as well as deformations due to the outer tube’s curvature, it was not possible to resolve near wall regions to extract the velocity field.

Due to the single camera setup, data was recorded on one plane at the time with two out of three velocity components available for each measurement. For side-holes and tip, due to missing volumetric information, drainage flow was computed through integration assuming axisymmetric flow from the side of the considered orifice to the respective orifice center. For all cases, the flow drained by the lighthouse tip cannula through each orifice, as fraction of total drained flow, is reported in Tables 2 and 3 for the ${0}^\circ$ and ${90}^\circ$ plane, respectively. The planar velocity data collected was also used to compute the Frobenius norm of the mean shear stress through the formula in Eq. (1).

1 $$\begin{aligned} \dot{\gamma } = \left[ \begin{array}{cc} \dfrac{\partial u}{\partial x} & \dfrac{1}{2}\left( \dfrac{\partial u}{\partial y} + \dfrac{\partial v}{\partial x}\right) \\ \dfrac{1}{2}\left( \dfrac{\partial u}{\partial y} + \dfrac{\partial v}{\partial x}\right) & \dfrac{\partial v}{\partial y} \\ \end{array}\right] \end{aligned}$$

## Results

### Drainage Performance

The first row of side-holes (A–B or A’–B’, for $0^\circ$ and ${90}^\circ$, respectively) drained more than the remaining two rows combined. Further, the drainage flow distribution did not seem to be affected by the different flow rate ratios *Q* analyzed: the first row of side-holes (A–B or A’–B’) drained more flow than the side-holes closer to the tip (E–F or E’–F’). For all the cases, the total drainage flow through the side-holes on the ${0}^\circ$ plane was higher than that through the side-holes on the ${90}^\circ$ plane. A shift in cannula position affected the side-hole fraction of drained flow, but not the drainage profile along each line of side-holes. Considering the fraction of flow drained by each line of side-holes (i.e. ACE and BDF), the difference between the two lines was always within 6.3% ($Q=1$, $Q_c =1.3$ L/min, $r_c={3.4}$ mm). The difference of drained flow between the two lines in the ${90}^\circ$ plane never exceeded 5.1% ($Q=1$, $Q_c = {1.3}$ L/min, $r_c={3.4}$ mm). For the ${0}^\circ$ plane, the centered case with the highest fraction of drained flow from the side-holes had $Q_c = {1.3}$ L/min and $Q_o = {2.6}$ L/min ($Q=0.5$). In the ${90}^\circ$ plane instead, the centered case with the highest drained flow had $Q_c = {1.3}$ L/min and $Q_o = {1.3}$ L/min ($Q=1$).

**Table 2:** Drainage fractions through side-holes and tip of the lighthouse tip cannula for ${0}^\circ$ plane and different drainage flow ($Q_c$), co-flow ($Q_o$), and lateral shift ($r_c$)

$r_c$ (mm)	$Q_c$ (L/min)	$Q_o$ (L/min)	*Q* (–)	A (%)	B (%)	C (%)	D (%)	E (%)	F (%)	Tip (%)
**0**	**1**.**3**	**1**.**3**	**1**	17.4	17.6	6.1	5.9	2.5	2.5	8.7
**0**	**1.3**	**2.6**	**0.5**	20.2	20.3	6.6	6.6	0.2	1.2	2.0
**0**	**2.6**	**1.3**	**2**	13.5	15.1	4.5	4.3	2.0	1.9	18.2
**0**	**2**.**6**	**2**.**6**	**1**	15.1	16.3	5.1	5.4	2.2	2.1	5.3
**1**.**32**	**1**.**3**	**1**.**3**	**1**	12.1	14.1	5.9	6.1	1.9	2.2	6.7
**1**.**32**	**1**.**3**	**2**.**6**	**0**.**5**	14.5	15.8	5.5	6.4	1.1	1.3	2.4
**1.32**	**2.6**	**1.3**	**2**	11.4	11.8	4.1	3.8	1.8	1.6	18.6
**1**.**32**	**2**.**6**	**2**.**6**	**1**	11.6	12.8	4.1	4.9	1.8	1.7	5.9
**3**.**4**	**1**.**3**	**1**.**3**	**1**	9.9	13.0	4.9	6.9	0.9	2.1	6.6
**3**.**4**	**1**.**3**	**2**.**6**	**0**.**5**	11.0	14.5	5.0	6.5	0.7	1.3	5.9
**3**.**4**	**2**.**6**	**1**.**3**	**2**	7.4	8.2	2.8	3.9	1.3	1.3	17.3
**3**.**4**	**2**.**6**	**2**.**6**	**1**	6.7	8.2	4.0	5.1	0.7	1.5	7.1

Bold was used to mark measured values while normal text was used to report estimated values (using axisymmetric flow assumption)

Flow rate ratio *Q* is defined as $Q = Q_c/Q_o$. Drainage fractions are given as percentages of total drainage flow ($Q_c$). Cases with velocity or shear stress data presented are underlined

**Table 3:** Drainage fractions through side-holes and tip of the lighthouse tip cannula for ${90}^\circ$ plane and different drainage flow ($Q_c$), co-flow ($Q_o$), and lateral shift ($r_c$)

$r_c$ (mm)	$Q_c$ (L/min)	$Q_o$ (L/min)	*Q* (–)	A’ (%)	B’ (%)	C’ (%)	D’ (%)	E’ (%)	F’ (%)	Tip (%)
**0**	**1**.**3**	**1**.**3**	**1**	10.9	11.5	4.2	4.7	1.2	1.5	4.9
**0**	**1.3**	**2.6**	**0.5**	12.8	12.7	2.7	2.9	0.2	0.7	2.3
**0**	**2.6**	**1.3**	**2**	8.6	9.1	3.3	3.3	1.3	1.3	17.4
**0**	**2**.**6**	**2**.**6**	**1**	10.8	11.3	4.0	4.4	0.7	1.3	3.8
**1**.**32**	**1**.**3**	**1**.**3**	**1**	9.6	9.8	3.8	3.9	1.5	1.5	3.9
**1**.**32**	**1**.**3**	**2**.**6**	**0**.**5**	10.7	11.1	3.2	3.2	0.6	0.6	0.1
**1.32**	**2.6**	**1.3**	**2**	6.9	7.3	2.7	2.9	1.1	1.2	18.5
**1**.**32**	**2**.**6**	**2**.**6**	**1**	8.9	9.7	3.1	3.0	1.0	0.9	5.3
**3**.**4**	**1**.**3**	**1**.**3**	**1**	9.4	6.2	4.8	3.0	1.4	1.2	7.6
**3**.**4**	**1**.**3**	**2**.**6**	**0**.**5**	9.8	7.0	4.4	3.6	0.3	0.4	5.1
**3**.**4**	**2**.**6**	**1**.**3**	**2**	6.8	4.5	3.1	2.4	1.2	1.0	17.5
**3**.**4**	**2**.**6**	**2**.**6**	**1**	8.1	4.3	3.7	2.6	0.9	1.0	5.5

Bold was used to mark measured values while normal text was used to report estimated values (using axisymmetric flow assumption)

Flow rate ratio *Q* is defined as $Q = Q_c/Q_o$. Drainage fractions are given as percentages of total drainage flow ($Q_c$). Cases with velocity or shear stress data presented are underlined

When the cannula was shifted in a position closer to the wall, reduced drainage capacity was observed for the side-holes on the *narrow side* (A, C, and E). Interestingly, also the side-holes on the ${90}^\circ$ plane saw a slight reduction of drainage flow. However, for all cases, the proximal side-hole (A) drained the highest fraction of fluid among the holes on the *narrow side*. For the case having $Q_c$ of 1.3 L/min and $Q_o$ of 2.6 L/min ($Q = 0.5$), hole A showed the greatest reduction of fractional drainage flow (9.2%) when $r_c$ was increased from 0 to 3.4 mm. For the same case, ($Q =0.5$), the total drainage fraction for the line of side-holes on the *narrow side* decreased by 10.3%. The line of side-holes on the *wide side* of the cannula (B, D, and F) also displayed a reduction in drainage flow although maintaining a similar distribution within the line of side-holes as compared to their respective centered case.

As the flow rate ratio was altered, the fractional end-hole drainage changed. In particular, cases with $Q =0.5$ ($Q_c$ of 1.3 L/min, $Q_o$ of 2.6 L/min) consistently showed the lowest fraction of drainage via the end-hole independent of the cannula shift, with values increasing when moving from the centered to most shifted configurations. This case ($Q=0.5$) was also characterized by the first row of side-holes (A–B or A’–B’) draining the largest fraction of total drainage flow in their respective plane. The drainage flow through the tip of the lighthouse tip cannula never exceeded 18.6% of the total drainage flow rate, recorded for $Q_c =2.6$ L/min, $Q_o=1.3$ L/min ($Q = 2$), and $r_c=1.32$ mm.

**Fig. 3:**
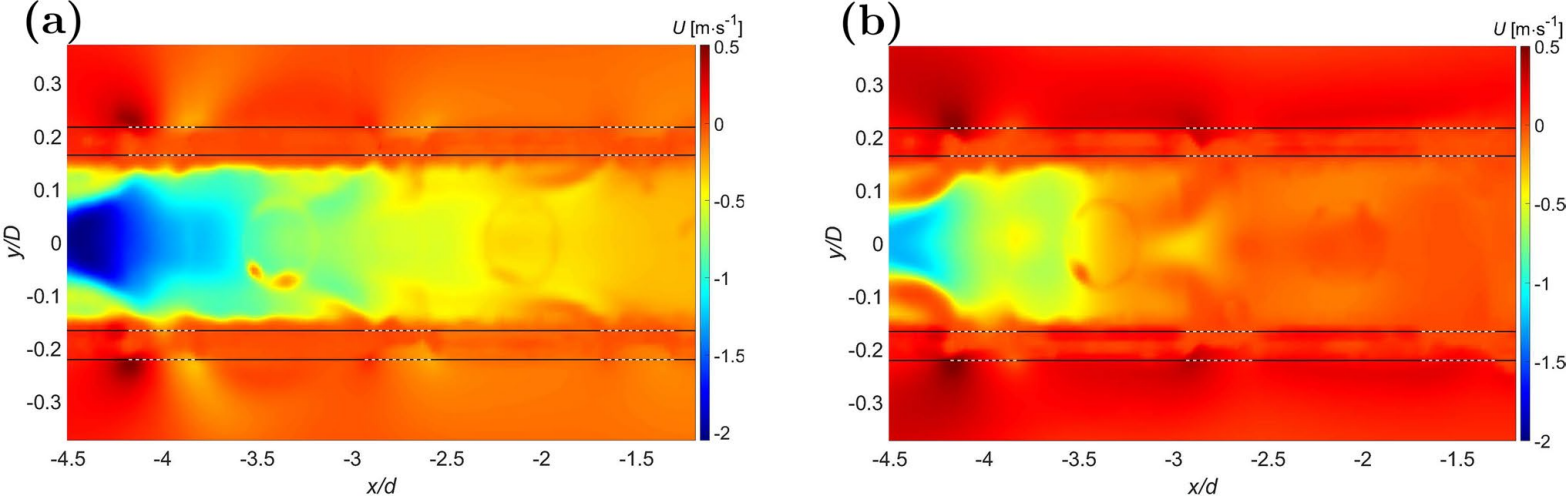
Detailed view of the averaged axial velocity component *U* of the centered ($r_c=0$ mm) lighthouse tip cannula for **a** cannula flow $Q_c={2.6}$ L/min and co-flow $Q_o={1.3}$ L/min ($Q = 2$), **b** $Q_c={1.3}$ L/min and $Q_o={2.6}$ L/min ($Q = 0.5$) on the ${0}^\circ$ plane. The continuous line marks the position of the cannula walls. The dashed line marks the position of the side-holes of the lighthouse tip cannula.

### Flow Characteristics Comparison

Compared to a blunt tip cannula, where all drainage occurs via the end-hole at the tip, the lighthouse tip cannula generated decidedly different flow field patterns. As visible in Fig. 3, where the side-holes are located, the flow outside the cannula was bent and entered the cannula forming a Y-shaped inflow, as first reported by Rauh et al. [13]. Further, Fig. 3 shows how the flow entering from opposing side-holes created an area with higher velocity in the core region of the cannula, with magnitude increasing with the vicinity to the proximal row of side-holes.

**Fig. 4:**
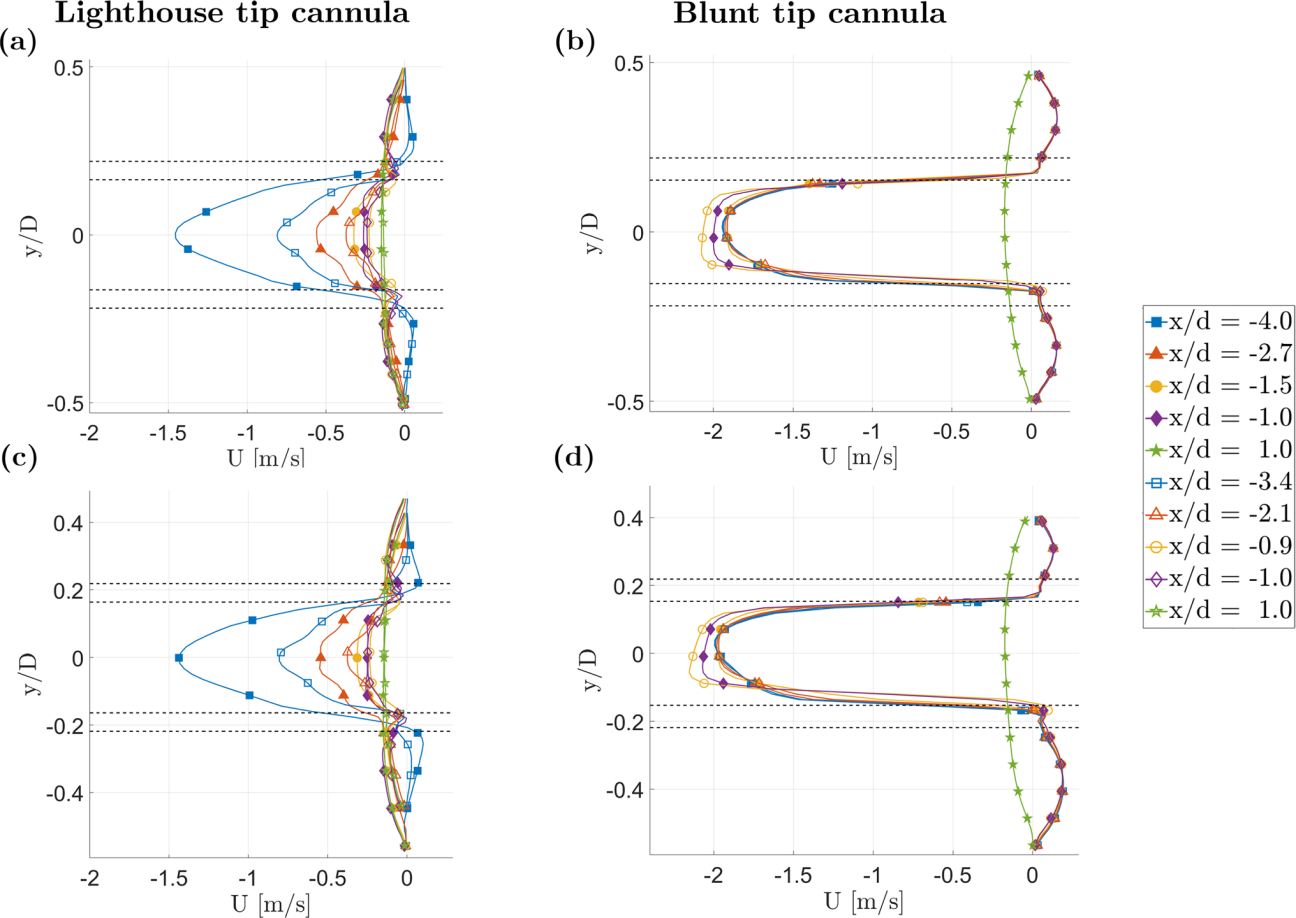
Averaged axial velocity component *U* for the lighthouse (left column **a**, **c**) and blunt (right column **b**, **d**) tip cannula for cannula drainage flow $Q_c={2.6}$ L/min, and co-flow $Q_o={1.3}$ L/min ($Q = 2$). For **a** and **b** the lateral shift $r_c = {0}$ mm and for **c** and **d** $r_c = {1.32}$ mm. *x*/*d* indicates the distance from the cannula tip normalized by the external cannula diameter. Dashed black lines mark the position of the cannula walls. Data from the ${0}^\circ$ plane is marked with filled symbols while empty symbols are data from the ${90}^\circ$ plane. Blunt tip cannula data was extracted at similar locations to the lighthouse tip cannula on a single plane but reported with filled and empty symbols to ease comparison.

Based on data from the ${0}^\circ$ and ${90}^\circ$ planes, Fig. 4 shows the velocity profiles of the axial component *U* at specific locations along the cannula; three across the center of the side-holes and two extracted symmetrically 8 mm before and after the cannula tip, as schematically shown in Fig. 1d for the ${0}^\circ$ plane. The velocity profiles for the blunt tip cannula were extracted at the same position as used for the lighthouse tip cannula, with data from the ${0}^\circ$ plane represented by filled symbols and data of the ${90}^\circ$ plane by empty symbols. Compared to the blunt tip cannula having the same velocity magnitude along its length, the lighthouse tip cannula displayed generally lower values of axial velocity (Fig. 4). The axial component of the velocity *U* in the lighthouse tip cannula experienced a rapid decrease of the magnitude while moving from the proximal side-holes (A–B) towards the tip. Near holes A–B and A’–B’ ($-3.5<x/d<-4.5$), the lighthouse tip cannula created strong spatial gradient in the axial direction for the axial velocity component *U*. For side-holes A–B, this is visible in Fig. 3 where the region with $-3.5<x/d<-4.5$ displays a fast spatial increase in velocity within the cannula core, higher than for the blunt tip cannula where velocities did not substantially change at the different locations investigated, Fig. 4b and d. Further, the plug like profile observed in the blunt tip cannula was disrupted by the presence of the side-holes in the lighthouse tip cannula. Inside the lighthouse tip cannula, when moving from the tip along the negative *x* direction, the velocity magnitude increased and the velocity profile became more parabolic.

The green profile in Fig. 4, acquired at $x/d = 1$, presented similar values of axial velocity component (*U*) when comparing lighthouse and blunt tip cannula. The symmetry in the velocity profile was broken for both designs when the cannula was laterally shifted. The location of maximum velocity of the extracted profile moved with the cannula shift for the blunt tip cannula and opposite to it for the lighthouse tip cannula, as visible in Fig. 4c and d.

On both ${0}^\circ$ and ${90}^\circ$ planes, when $Q_c = {2.6}$ L/min and $Q_o = {1.3}$ L/min ($Q = 2$), the lighthouse tip cannula generated a reversed flow region between the cannula and outer tube in a position between the first and last side-holes of each line. This is demonstrated by the negative axial velocity values in the profiles across the middle of the side-holes rows C–D and E–F (red and yellow lines in Fig. 4, respectively). Reversed flow was not observed across A–B (blue line) or A’–B’, where the velocity outside the cannula body had a positive or null value. On the ${0}^\circ$ plane, the starting location of the reverse flow fluctuated between $x/d = -3.4$ and $-2.5$ for all flow rate ratios except than $Q =2$ ($Q_c={2.6}$ L/min and $Q_o = {1.3}$ L/min). The starting location of reverse flow on the ${90}^\circ$ plane instead fluctuated between $x/d = -2.9$ and $-2.3$, still with the exception of the cases with *Q* = 2 ($Q_c=$ 2.6L/min and $Q_o = {1.3}$ L/min). When the cannula was shifted, for the ${0}^\circ$ plane, the reversed flow region was reduced on the *narrow side* whereas still observable on the *wide side*, Fig. 4c for $r_c$ of 1.32 mm. No considerable difference of the recirculating region on the sides of the cannula was observed on the ${90}^\circ$ plane when the cannula was shifted.

**Fig. 5:**
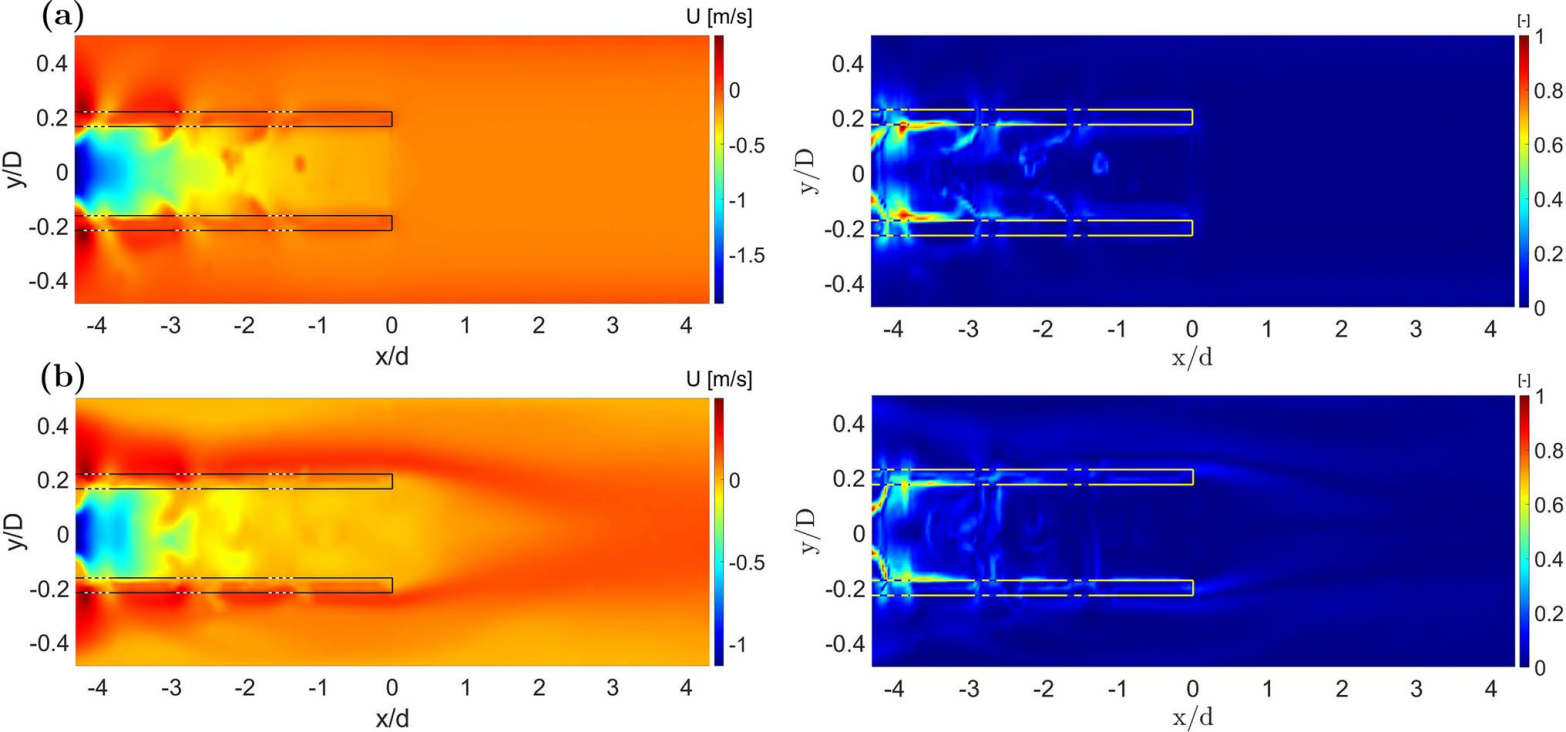
Averaged axial velocity component *U* (left) and Frobenius norm of the shear stress matrix normalized with maximum value in the domain (right) of the centered lighthouse tip cannula on the ${0}^\circ$ plane for **a** cannula drainage flow $Q_c = {2.6}$ L/min and co-flow $Q_o = {1.3}$ L/min ($Q=2$) and **b** $Q_c ={1.3}$ L/min and $Q_o = {2.6}$ L/min ($Q=0.5$). *x*/*d* is the distance from the cannula tip normalized by the external cannula diameter. The continuous line marks the position of the cannula walls. The dashed line marks the position of the side-holes of the lighthouse tip cannula

For the case with $Q_c={1.3}$ L/min and co-flow $Q_o={2.6}$ L/min ($Q = 0.5$) a region of near stagnant flow formed in front of the cannula tip, as visible to the left of Fig. 5b. This was observed in both the $0^\circ$ and $90^\circ$ planes and is similar to the wake forming after a bluff body. Here, the vessel flow formed a confined region of near stagnant flow reaching up to 4 cannula diameters ($x/d \approx 4$) downstream of the tip, consistent with results reported in literature for similar flow configuration of backward facing step (BFS) [25]. For this configuration ($Q = 0.5$), when the cannula was shifted from the centered to the most lateral position the flow on the *wide side* continued along the cannula while it was gradually interrupted on the *narrow side*. This broke the symmetry in the region of near stagnant flow and allowed marginally better drainage from the tip of the lighthouse tip cannula in the most shifted configuration.

### Shear Stress Characteristics

**Fig. 6:**
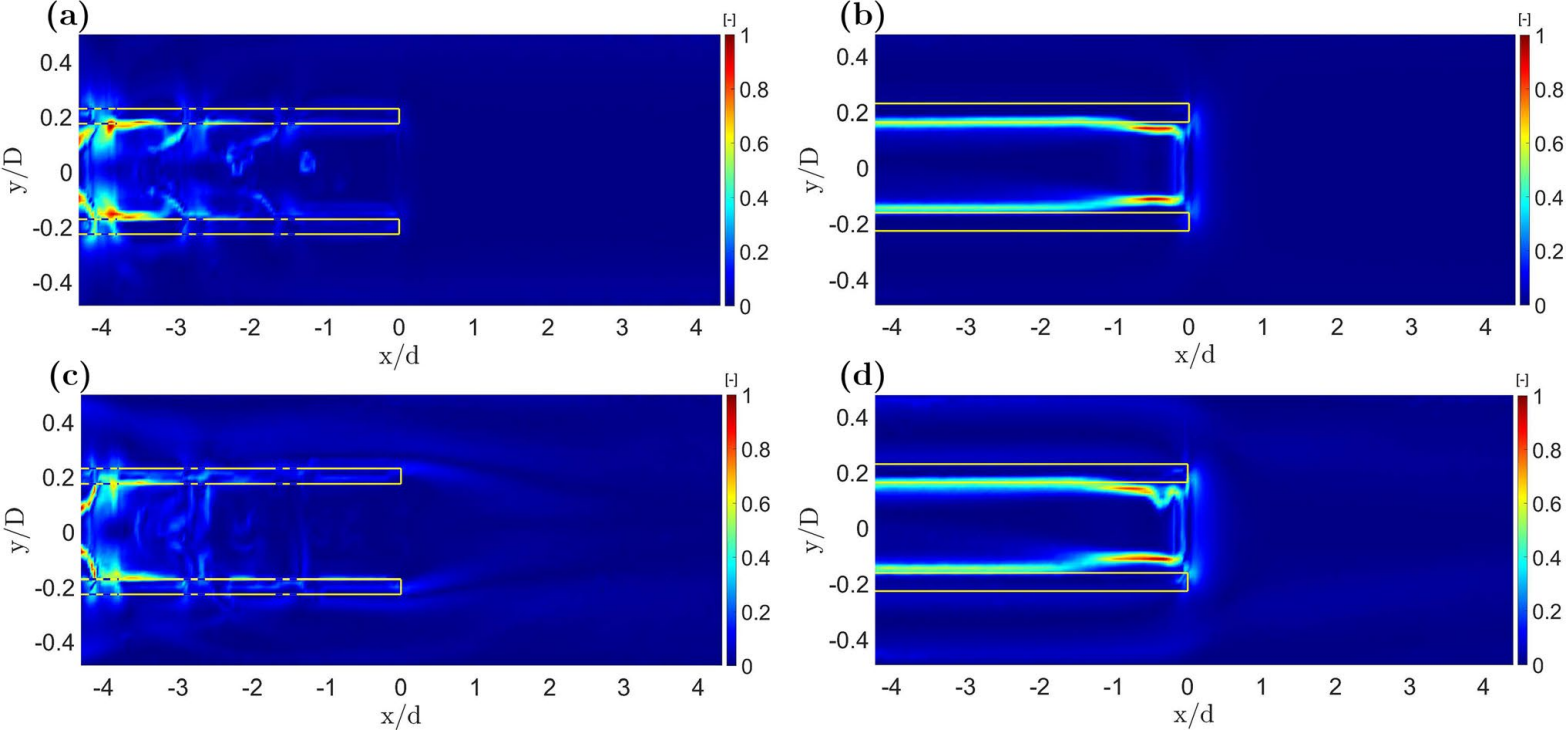
Frobenius norm of the shear stress matrix normalized with maximum value of shear in the domain for centered lighthouse tip on the ${0}^\circ$ plane (**a, c**) and blunt tip cannula (**b, d**). Panels **a** and **b** have cannula drainage flow $Q_c={2.6}$ L/min and co-flow $Q_o={1.3}$ L/min ($Q=2$) while **c** and **d** have $Q_c ={1.3}$ L/min and $Q_o={2.6}$ L/min ($Q =0.5$). *x*/*d* is the distance from the cannula tip normalized by the external cannula diameter. The continuous line marks the position of the cannula walls. The dashed line marks the position of the side-holes of the lighthouse tip cannula

The computed Frobenius norm of the image sequence, normalized with the maximum shear stress in time, for cases with a centered cannula and $Q = 2$ or 0.5 are shown in Fig. 6 for the blunt tip and the lighthouse tip cannula on the ${0}^\circ$ plane. The presence of the side-holes altered the flow behavior and consequently the shear stress levels experienced by the flow, as visible in Fig. 6a and c for the lighthouse design and Fig. 6b and d for the blunt design, respectively. Although not shown here, the shear stress levels in the ${90}^\circ$ plane had similar distribution but lower magnitude than on the ${0}^\circ$ plane. The maximum and mean shear experienced using the lighthouse tip cannula were generally lower than the maximum and mean levels of shear experienced while using the blunt tip cannula. However, the blunt tip cannula was found to have less extended areas of shear stress higher than the average stress when compared to the lighthouse design. For both designs, although the maximum values differed, shear stress levels 20% higher than the average value were not recorded in more than 27% of the domain. This indicates localized maximum shear stress for both geometries and shear stress values generally far from the mean shear stress recorded in the domain.

Between blunt tip and lighthouse tip cannula, the shear stress levels varied and areas of maximum shear were localized in different specific regions. The blunt tip cannula induced stresses inside its lumen along the wall with the highest value registered close to the tip. The lighthouse tip cannula showed shear stresses distributed all along the side-holes region. Higher values were attained closer to the row of proximal side-holes (A–B), whereas lower but still observable levels of shear were observed in the next two rows of side-holes (C–D and E–F). Shear stress on the ${90}^\circ$ plane had a similar behavior with values around the first row of side-holes (A’–B’) generally higher than values around the next two rows (C’–D’ and E’–F’). The magnitude of the shear stresses on the ${90}^\circ$ plane was generally lower than on the ${0}^\circ$ plane for the same location, flow configuration, and cannula position. The interaction between fluid inside the cannula and entering from the side-holes created a shear layer and a region of near stagnant flow to the left of each side-hole, as visible in Fig. 6a and c. In the shear layer region, characterized by light blue color in Fig. 6, the fluid experienced higher shear stress levels than in the surrounding domain. Around the tip of the lighthouse tip cannula considerably lower values of shear stress than in other locations of the cannula were observed, in contrast to the shear stresses observed in the blunt tip cannula. When tilting the cannula, the general distribution of shear stress did not change with similar trends to the centered case. Mean values did not differ by more than 15% between the centered and the most shifted case.

## Discussion

Two rigid cannula designs, a lighthouse and a blunt tip cannula, were investigated to characterize the drainage performance through each orifice and the level of shear stress originated during drainage. Studies were performed both with a centered ($r_c = {0}$ mm) as well as a shifted cannula ($r_c$ of 1.32 or 3.4 mm) for different flow rate ratios.

The lighthouse tip cannula had the highest drainage in the proximal row of side-holes. The fraction of drained flow reduced when moving from the proximal side-holes to the cannula tip. The general trend of the first row of side-holes in each plane (A-B or A’–B’ for ${0}^\circ$ and ${90}^\circ$ plane, respectively) draining notably more than the remaining two side-holes rows combined is consistent with other experimental [15, 26] and numerical studies [13, 18]. When compared to a lighthouse tip cannula the blunt tip cannula induced higher levels of shear stress for a similar flow configuration and cannula position.

Different flow rate ratios as well as the position of the cannula within the outer tube affected the drainage performance. This feature was driven by high spatial gradients of velocity and pressure in the region around the proximal row of side-holes (A–B). The gradients decreased while moving from the proximal side-holes towards the tip. When the lighthouse tip cannula was shifted towards the outer wall, the holes on the *narrow side* showed a decrease in the fractional drainage flow. However, on the ${0}^\circ$ plane, the longitudinal distribution along the lines of side-holes was maintained and the proximal side-holes (A and B) still drained the highest volume flow fraction. On the ${90}^\circ$ plane, the first row of side-holes (A’–B’) drained more than the next two rows for all the cases. But A’ and B’ combined had generally lower drainage than A and B, even as the cannula moved to more shifted configurations. Although this may depend on the position of A’ and B’, located longitudinally after A and B when moving from side-holes A-B toward the tip. As A’ and B’ are positioned after the location of the proximal side-holes, drainage fraction across A’–B’ will inherently be lower than across A–B.

Switching from a lighthouse to a blunt tip cannula created alterations of the drainage performance, flow field characteristics and shear stress levels. Between the two cannula designs, the lighthouse design generally induced lower axial velocities when compared to a blunt tip cannula. With the exception of the region right after the first row of side-holes ($x/d<-4$ in Figs. 5 and 6), where the two streams entering via the side-holes A and B formed a Y-shaped inflow structure in line with the results reported by Rauh et al. [13]. In this region the axial velocity (*U*) was higher in the lighthouse tip design when compared to the blunt tip cannula. This region also showed higher degree of spatial velocity gradients and shear stresses inside the lighthouse tip cannula when compared to the blunt tip cannula.

Regarding the general distribution of shear stresses, the higher recorded values were located near the inner walls of both cannulae, with the lighthouse tip cannula concentrating the highest levels of shear stress in the vicinity of proximal row of side-holes (A–B) on the ${0}^\circ$ plane. The stream entering the side-holes created a shear layer followed by a zone of near stagnant flow to the left of each side-hole. Instead, the blunt tip cannula experienced the higher values closer to its tip. For the blunt design, the case with flow rate ratio *Q* = 1 ($Q_c={1.3}$ L/min and $Q_o={1.3}$ L/min) produced the lowest maximum and mean values of shear stress in the domain for all considered positions. The maximum and mean shear stress values in the lighthouse design were similar on both investigated planes. The minimal values of shear stresses were recorded for $\textit{Q} = 1$ ($Q_c= {1.3}$ L/min and $Q_o = {1.3}$ L/min) on the ${0}^\circ$ plane while for $\textit{Q} = 0.5$ ($Q_c= {1.3}$ L/min and $Q_o = {2.6}$ L/min) on the ${90}^\circ$ plane. The maximum of the Frobenius norm of the shear stress matrix was 4.8 and 3.3 Pa for the blunt and lighthouse tip cannula, respectively. For the same flow rate configuration, the blunt tip cannula generally induced higher values of shear stress than the lighthouse tip cannula, in agreement with previous publications [11, 12]. This trend was retained also when the cannulae were in the shifted configurations.

The results presented in this study were obtained using water as the fluid medium. Despite being a fluid with lower viscosity and different rheological properties compared to blood, water was preferred over a non-Newtonian blood analogue fluid due to the reduced complexities in guaranteeing controlled fluid properties before, during, and after the experiments. An important factor to consider when data are collected also to be used for validation of numerical studies. Using a more viscous fluid as blood, and scaling the velocity through dynamic similarity, will produce higher dissipation and result in a reduction of the size of the near stagnant region. However, the higher dissipation may produce an increase of the shear stress levels observed. Further, effects of the fluid rheology are less critical in the area of interest, in this study around the side-holes, where shear rates are high enough to eliminate non-Newtonian effects [14].

For clinical use, flexible cannulae are needed for anatomical and safety reasons. However, flexibility creates difficulties in maintaining a centered position of the cannula within the vessel. To reduce complexity, we used rigid cannulae shifted towards the vessel wall in this study. This approach allowed the characterization of the fluid structures, drainage performance, and shear stress forming the basis for the flexible cannula flow situation in a more complex vessel geometry. When used clinically, the blunt tip cannula assessed in this work has a stented extension protruding forward from the tip [27]. This stainless steel mesh expands towards the vessel wall and prevents vascular collapse [28]. This cannula design provides high drainage [15, 28]. However, detailed flow structures in the tip region with the applied stent have not previously been investigated.

## Limitations

The assumption of axisymmetric flow through the side-holes, necessary due to the planar and not volumetric nature of the data collected, allowed the calculation of the fractional drainage mass flow rates through each orifice of the two cannula designs for different flow rate ratios and geometrical configurations. This assumption, the maximum velocity across the side-holes region, and the data scarcity near the walls led to differences in the computed drainage flow through each orifice when compared to a numerical simulation ran for the centered case and flow rate ratio $Q=2$. The simulation results also showed the axisymmetric assumption to not be correct [14]. In the experiments, the drainage flow on the ${0}^\circ$ plane was similar in the first and last row of side-holes (A–B and E–F, respectively) but was underestimated in the region of the second row of side-holes (C–D). Instead, due to lower flow rates across the side-holes on the ${90}^\circ$ plane, values of drainage flow were closer to the numerical results reported by Fiusco et al. [14]. For the dynamically similar case investigated numerically ($Q = 2$), when experimental and numerical results were compared, the estimated drainage flow through the side-holes did not differ by more than 17% on the ${0}^\circ$ plane and 3.6% on the ${90}^\circ$ plane, respectively. The maximum discrepancy on the ${0}^\circ$ plane was recorded in the second row of side-holes (C and D) where highly unsteady flow was reported by Fiusco et al. [14].

Near wall properties can be challenging to obtain with standard PIV techniques. In this study, the presence of curved surfaces prevented quantitative assessments. Another limitation was the drainage performance of the lighthouse tip cannula which was only analyzed on isolated cross-planes through six of the twelve holes, thus neglecting possible volumetric effects of the excluded side-holes in the observed flow dynamics. Further, only having access to planar PIV provided information of two out of the three velocity components during the experiments. This may lead to underestimation of the total shear stress levels experienced by the flow due to the missing velocity gradient information important to compute the full Frobenius norm.

The pulsatility present in the IVC was not considered in this study due to the intention of reducing complexity. Although blood flow in the IVC is pulsatile, a previous numerical study by our group investigating a model including IVC, superior vena cava, and right atrium showed minimal differences with respect to mean velocities in the IVC when inflow conditions were changed from steady to pulsatile [17].

Finally, this study did not consider flexibility of either cannula or containing vessel. Consequently, removing possible fluid–structure interactions between cannula, the fluid and the containing vessel. However, this simplification enabled the investigation of the fundamental fluid structures created by each cannula design in isolation and without the inclusion of effects originating by varying position or geometrical characteristics of the cannula.

## Conclusions

This work experimentally investigated drainage performance of rigid models of cannulae geometries used in ECMO, blunt and lighthouse tip (single-stage). The blunt tip cannula can only drain via the single end-hole. The lighthouse tip cannula had the highest drainage via the proximal side-holes, with drainage gradually decreasing in each row of side-holes while approaching the tip. When the lighthouse tip cannula was shifted towards the vessel wall, no major changes were observed in the drainage flow distribution through the lines of side-holes. However, occlusion gradually reduced the fractional flow drained via side-holes on the *narrow side* while side-holes on the *wide side* retained the highest drainage flow. In the lighthouse tip cannula the decreasing drainage distribution originated from a pressure imbalance between the outside and inside of the cannula. The pressure imbalance was highest near the proximal side-holes and gradually decreased while moving towards the tip. The high drainage flow and shear stresses recorded in the side-holes region, coupled with the regions of stagnant flow observed near side-holes and tip can increase the potential risk for thrombus formation in these sections of the cannula. Finally, higher levels of mean shear stress were recorder for the blunt tip cannula regardless of flow rate ratio or cannula shift, with maximum values near the blunt tip.

## Supplementary Information

Below is the link to the electronic supplementary material.

Supplementary file1 (PDF 1015 KB)

Supplementary file2 (ZIP 41,511 KB)

## Data Availability

The averaged velocity field data are available with the supplemental material. Instantaneous velocity data, as well as other data supporting the findings of this study, are available from the corresponding author upon reasonable request.

## Footnotes

1 Dimensions of cannula used in ECMO refers to outer diameter and is reported in French ($3\,\textrm{Fr} = {1}\,{\textrm{mm}}$). The length in centimeters refers to the maximum distance the tip can be advanced into a vessel (*insertion length*).

## References

[1] Hill, J. D., et al. Prolonged extracorporeal oxygenation for acute post-traumatic respiratory failure (shock-lung syndrome)—use of the Bramson membrane lung. *New Engl. J. Med.* 286:629–634, 1972. 10.1056/NEJM197203232861204. 5060491 10.1056/NEJM197203232861204

[2] Peek, J. G. K., et al. Efficacy and economic assessment of conventional ventilatory support versus extracorporeal membrane oxygenation for severe adult respiratory failure ([cesar]): a multicentre randomised controlled trial. *The Lancet*. 374(9698):1351–63, 2009. 10.1016/S0140-6736(09)61069-2. 10.1016/S0140-6736(09)61069-2 19762075

[3] Extracorporeal Life Support Organization, 2024 ecls international summary of statistics (2028). https://www.elso.org/registry/internationalsummaryandreports/internationalsummary.aspx

[4] Frenckner, B., M. Broman, and M. Broomé. Position of draining venous cannula in extracorporeal membrane oxygenation for respiratory and respiratory/circulatory support in adult patients. *Crit. Care*. 22(163) 2018. 10.1186/s13054-018-2083-08. 10.1186/s13054-018-2083-0 PMC6003129 29907121

[5] Costantini, S., M. Belliato, F. Ferrari, G. Gazzaniga, M. Ravasi, M. Manera, M. E. De Piero, A. Curcelli, A. Cardinale, and R. Lorusso. A retrospective analysis of the hemolysis occurrence during extracorporeal membrane oxygenation in a single center. *Perfusion.* 1–13, 2022. 10.1177/02676591211073768. 10.1177/02676591211073768 35225087

[6] Materne, L. A., et al. Hemolysis in patients with extracorporeal membrane oxygenation theraphy for severe acute respiratory distress syndrome—a systematic review of the literature. *J. Med. Sci.* 18(8):1730–1738, 2021. 10.7150/ijms.50217. 10.7150/ijms.50217 PMC7976579 33746589

[7] Sen, A., et al. Adult venovenous extracorporeal membrane oxygenation for severe respiratory failure: Current status and future perspectives. *Ann. Card. Anaesth.* 19:97–111, 2016. 10.4103/0971-9784.173027. 26750681 10.4103/0971-9784.173027 PMC4900379

[8] Vaquer, S., et al. Systematic review and meta-analysis of complications and mortality of veno-venous extracorporeal membrane oxygenation for refractory acute respiratory syndrome. *Annuals Intensive Care*. 7(51):1–2, 2017. 10.1186/s13613-017-0275-4. 10.1186/s13613-017-0275-4 PMC5429319 28500585

[9] Iacobelli, R., et al. Predictors of brain infarction in adult patients on extracorporeal membrane oxygenation: an observational cohort study. *Sci. Rep.* 11(3809), 2021. 10.1038/s41598-021-83157-5. 10.1038/s41598-021-83157-5 PMC7884423 33589664

[10] Grigioni, M., et al. Computational model of the fluid dynamics of a cannula inserted in a vessel: Incidence of the presence of side holes in blood flow. *J. Biomech.* 35(12):1599–612, 2002. 10.1016/s0021-9290(02)00231-2. 12445613 10.1016/s0021-9290(02)00231-2

[11] Goto, T., et al. Effect of inflow cannula side-hole number on drainage flow characteristics: Flow dynamic analysis using numerical simulation. *Perfusion*. 33(8):649–655, 2018. 10.1177/0267659118782246. 29956567 10.1177/0267659118782246

[12] Park, J. Y. The need of slanted side holes for venous Cannulae. *Comput. Math. Methods Med.* 2012, 2012. 10.1155/2012/854938. 10.1155/2012/854938 PMC3265159 22291856

[13] Rauh, P., et al. Determination of local flow ratios and velocities in a femoral venous cannula with computational fluid dynamics and 4d flow-sensitive magnetic resonance imaging: A method validation. *Artif. Organs*. 45(5):506–515, 2021. 10.1111/AOR.13859. 33185904 10.1111/aor.13859

[14] Fiusco, F., F. Rorro, L. M. Broman, and L. Prahl Wittberg. Numerical and experimental investigation of a lighthouse tip drainage cannula used in extracorporeal membrane oxygenation. *Artif. Organs*, 2022. 10.1111/aor.14421. 10.1111/aor.14421 PMC10092507 36227654

[15] Broman, L. M., L. Prahl Wittberg, C. J. Westlund, M. Gilbers, L. Perry da Câmara, J. Westin, F. S. Taccone, M. V. Malfertheiner, M. Di Nardo, J. Swol, L. Vercaemst, N. A. Barrett, F. Pappalardo, J. Belohlavek, T. Müller, M. Belliato, and R. Lorusso. Pressure and flow properties of cannulae for extracorporeal membrane oxygenation II: Drainage (venous) cannulae. *Perfusion (United Kingdom)*. 34(1–suppl):65–73, 2019. 10.1177/0267659119830514. 10.1177/0267659119830514 30966909

[16] Wong, K. C., et al. Effect of inflow cannula tip design on potential parameters of blood compatibility and thrombosis. *Int. J. Artif. Organs*. 37(12):875–87, 2014. 10.5301/ijao.5000361. 25450321 10.5301/ijao.5000361

[17] Parker, L. P., A. Svensson Marcial, T. B. Brismar, L. M. Broman, and L. Prahl Wittberg. Impact of altered vena cava flow rates on right atrium flow characteristics. *J. Appl. Physiol.* 132(5):1167–1178, 2022. 10.1152/japplphysiol.00649.2021. 35271411 10.1152/japplphysiol.00649.2021 PMC9054263

[18] Vatani, A., et al. Improved drainage cannula design to reduce thrombosis in veno-arterial extracorporeal membrane oxygenation. *Am. Soc. Artif. Intern. Organs*. 68(2):205–213, 2022. 10.1097/MAT.0000000000001440. 10.1097/MAT.0000000000001440 33883503

[19] Lemétayer, J., L. Mikael Broman, and L. Prahl Wittberg. Flow dynamics and mixing in extracorporeal support: A study of the return cannula. *Front. Bioeng. Biotechnol.* 9:1–2, 2021. 10.3389/fbioe.2021.630568. 10.3389/fbioe.2021.630568 PMC7902508 33644022

[20] Fulker, D., et al. Computational fluid dynamic analysis of the hemodialysis plastic cannula. *Artif. Organs*. 41(11):1035–1042, 2017. 10.1111/aor.12901. 28591486 10.1111/aor.12901

[21] Prince, M. R., et al. The diameter of the inferior vena cava and its implications for the use of vena caval filters. *Radiology*. 149(3):687–9, 1983. 10.1148/radiology.149.3.6647844. 6647844 10.1148/radiology.149.3.6647844

[22] Finnerty, N. M., et al. Inferior vena cava measurement with ultrasound: What is the best view and best mode? (2017). 10.5811/westjem.2016.12.32489 10.5811/westjem.2016.12.32489 PMC5391901 28435502

[23] Voelker, M. T., et al. Restrictive transfusion practice during extracorporeal membrane oxygenation therapy for severe acute respiratory distress syndrome. *Artif. Organs*. 39:374–378, 2015. 10.1111/aor.12385. 25349127 10.1111/aor.12385

[24] Cherry, E. M., and J. K. Eaton. Shear thinning effects on blood flow in straight and curved tubes. *Phys. Fluids*. 25(7), 2013. 10.1063/1.4816369.

[25] Chen, L., et al. A review of backward-facing step (BFS) flow mechanisms, heat transfer and control. *Therm. Sci. Eng. Prog.* 194–216, 2018. 10.1016/j.tsep.2018.04.004.

[26] Lindholm, J. A. Cannulation for veno-venous extracorporeal membrane oxygenation. *J. Thorac. Dis.* 10:606–612, 2018. 10.21037/jtd.2018.03.101. 10.21037/jtd.2018.03.101 PMC5911563 29732177

[27] Smartcannula llc. ECMO smart cannula^®^ specifications (2021). http://www.smartcanula.com/index.php?option=com_content&task=view &id=43 &Itemid=81. [Online; last accessed September 2022]

[28] Jegger, D., et al. Using computational fluid dynamics to evaluate a novel venous cannula (Smart cannula®) for use in cardiopulmonary bypass operating procedures. *Perfusion*. 22:257–265, 2007. 10.1177/0267659107083657. 18181514 10.1177/0267659107083657

