# Supplementary material for: Performance Comparison of Centered and Tilted Blunt and Lighthouse Tip Cannulae for Drainage in Extracorporeal Life Support
Source: Cardiovasc Eng Technol. 2025 Feb 10;16(2):238–50. doi: 10.1007/s13239-024-00770-x (PMC11933157; doi:10.1007/s13239-024-00770-x)
Supplement: Supplementary file 1 — Supplementary file1 (PDF 1015 KB) [file 13239_2024_770_MOESM1_ESM.pdf]

Supplemental Material S1

Average velocity fields

In the folder **avg_velocity** can be found the average values of the two recorded velocity components for all the cases acquired with blunt and lighthouse tip cannula. The data is divided according to the cannula lateral shift (r_c) in the outer tube. The main folder contains three sub-folders named:

- **rc_0_mm** includes data for cases with $r_c = 0$ mm (*i.e.* centered cannula)
- **rc_1.32_mm** includes data for cases with $r_c = 1.32$ mm (*i.e.* cannula shifted by 1.32 mm in outer the tube)
- **rc_3.4_mm** includes data for cases with $r_c = 3.4$ mm (*i.e.* cannula shifted by 3.4 mm in outer the tube)

Within each sub-folder are then collected the data acquired for lighthouse tip cannula, for both the 0° and 90° planes, as well as the blunt tip cannula. The different collected data are included in folders with names given depending on the case from which the data was obtained. For example:

- *Lighthouse_0_Qc_1.3_Lmin_Qo_2.6_Lmin_rc_1.32_mm*
  is a folder that contains data of the lighthouse tip cannula on the 0° plane with cannula flow (Q_c) of 1.3 L/min, outer flow (Q_o) of 2.6 L/min, and lateral shift (r_c) of 1.32 mm
- *Lighthouse_90_Qc_2.6_Lmin_Qo_1.3_Lmin_rc_1.32_mm*
  is a folder that contains data of the lighthouse tip cannula on the 90° plane with cannula flow (Q_c) of 2.6 L/min, outer flow (Q_o) of 1.3 L/min, and lateral shift (r_c) of 1.32 mm
- *Blunt_Qc_2.6_Lmin_Qo_1.3_Lmin_rc_3.4_mm*
  is a folder that contains data of the blunt tip cannula with cannula flow (Q_c) of 2.6 L/min, outer flow (Q_o) of 1.3 L/min, and lateral shift (r_c) of 3.4 mm

Within each folder containing the data of a single case are present four files. These files are the following:

- **x_adim.csv**
- **y_adim.csv**
- **U_ms.csv**
- **V_ms.csv**

The first two (**x_adim.csv** and **y_adim.csv**) contain information of the x-coordinate divided by the cannula external diameter (d=8 mm; **x_adim** = x/d) and y-coordinate divided by the inner diameter of the outer tube (D=18.3 mm; **y_adim** = y/D). The other two files (**U_ms.csv** and **V_ms.csv**) contain information of the velocity component in m/s along the x-coordinate (U) and along the y-coordinate (V), respectively.

The file structure used while saving the files, for each one of the four files, is explained in the following list.

- **x_adim.csv**: Values in the data array do not change when moving along a fixed column. However, values due change when moving along a fixed row. This ensures that each column will mark a point with a specific x-coordinate independent of the selected row (*i.e.* values [1 , 1] and [20 , 1] will be the same)
- **y_adim.csv**: Values in the data array do not change when moving along a fixed row. However, values due change when moving along a fixed column. This ensures that each row will mark a point with a specific y-coordinate independent of the selected column (*i.e.* values [1 , 1] and [1 , 20] will be the same)
- **U_ms.csv**: This data array contains information of the velocity along the x axis in m/s. To know the position where the velocity data was obtained, the values having the same location in the arrays of files **x_adim.csv** and **y_adim.csv** should be also extracted. These values will give the x- and y-coordinate of the horizontal velocity component (*i.e.* element [4 , 31] from **U_ms.csv** will have x and y-coordinate taken from element [4 , 31] of **x_adim.csv** and **y_adim.csv**, respectively.
- **V_ms.csv**: This data array contains information of the velocity along the y axis in m/s. Similarly to **U_ms.csv**, to obtain the coordinate information of where the value of the velocity component (*e.g.* [7 , 28]) was computed is necessary to extract the values of the x- and y-coordinate from element [7 , 28] of the **x_adim.csv** and **y_adim.csv** arrays, respectively.

The size of the array in the .csv data files represents the size of the planar velocity field map obtained after the adaptive PIV algorithm within DynamicStudio v.2015a (Dantec Dynamics, Skovlunde, Denmark) was applied on the collected image series.

Shear stresses on 90° plane

The Frobenius norm of the mean shear stress was computed with the formula equation below.

$$\dot{\gamma} = \begin{bmatrix} \dfrac{\partial u}{\partial x} & \dfrac{1}{2}\left(\dfrac{\partial u}{\partial y} + \dfrac{\partial v}{\partial x}\right) \\ \dfrac{1}{2}\left(\dfrac{\partial u}{\partial y} + \dfrac{\partial v}{\partial x}\right) & \dfrac{\partial v}{\partial y} \end{bmatrix}$$

The computed norm of the image sequence, normalized with the maximum shear stress in time, for cases with a centered lighthouse tip cannula on the 90° plane are displayed in Figure 1, Figure 2, Figure 3, and Figure 4. Each figure caption contains information about the flow ratio with which the data were collected. In the caption of all the figures Q_c is the value of cannula flow while Q_o is the value of the outer flow. The flow rate ratio Q is computed as the ratio of cannula to vessel flow (*i.e.* $Q = Q_c/Q_o$)

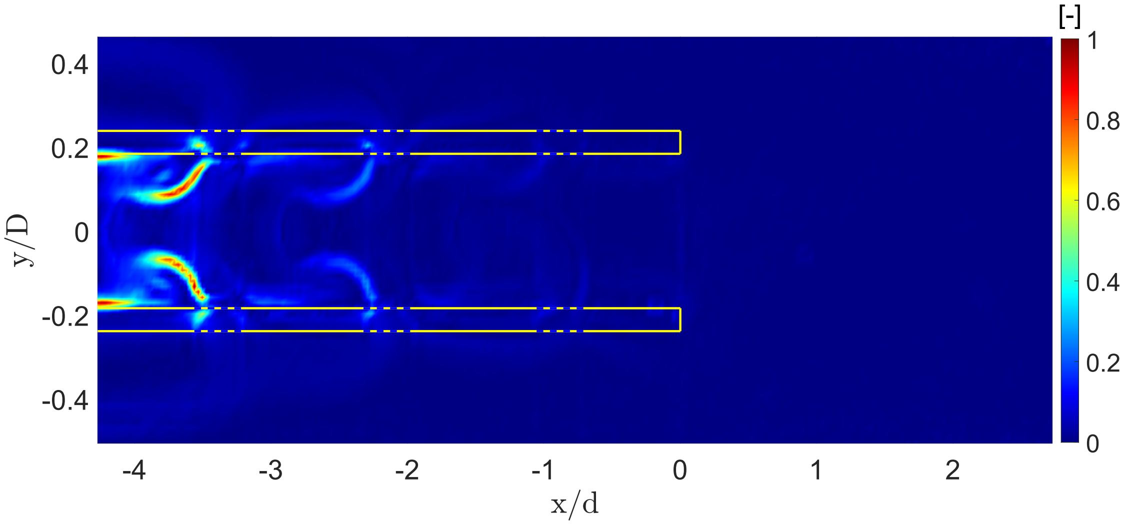

Figure 1: $Q_c = 1.3\,\mathrm{L/min}$ and $Q_o = 1.3\,\mathrm{L/min}$ ($Q = 1$)

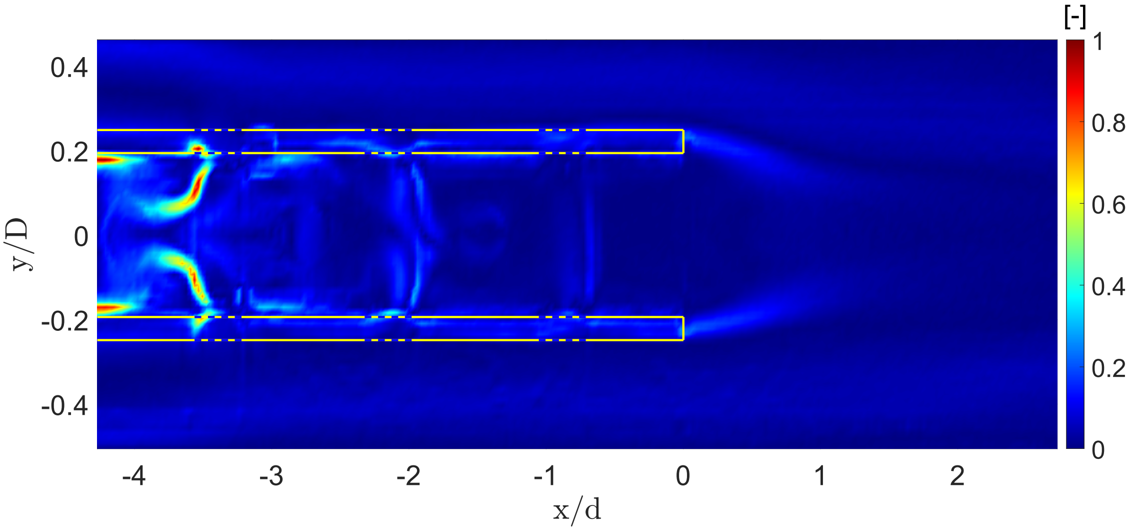

Figure 2: $Q_c = 1.3\,\mathrm{L/min}$ and $Q_o = 2.6\,\mathrm{L/min}$ ($Q = 0.5$)

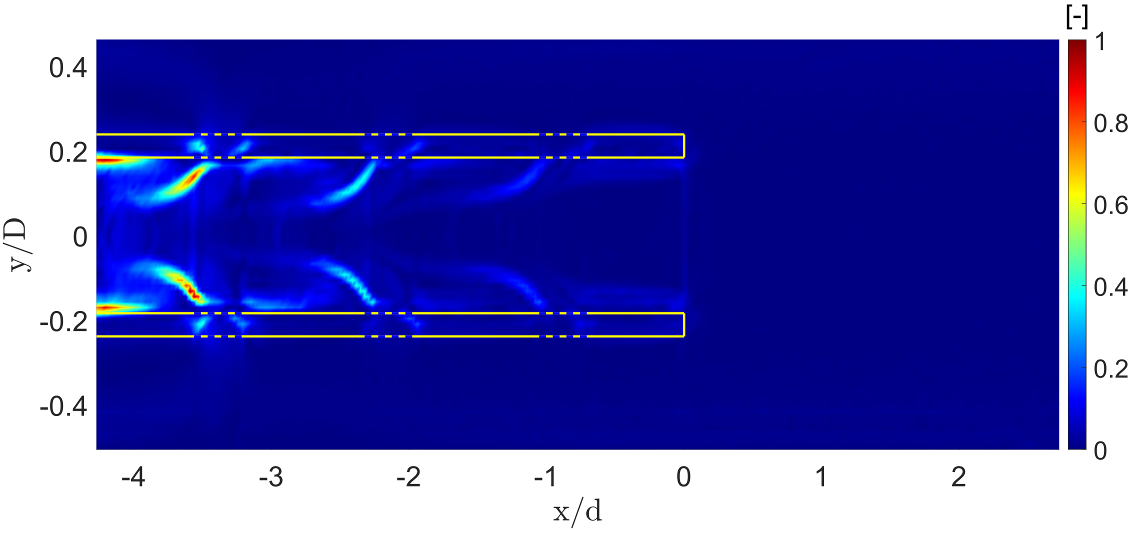

Figure 3: $Q_c = 2.6\,\mathrm{L/min}$ and $Q_o = 1.3\,\mathrm{L/min}$ ($Q = 2$)

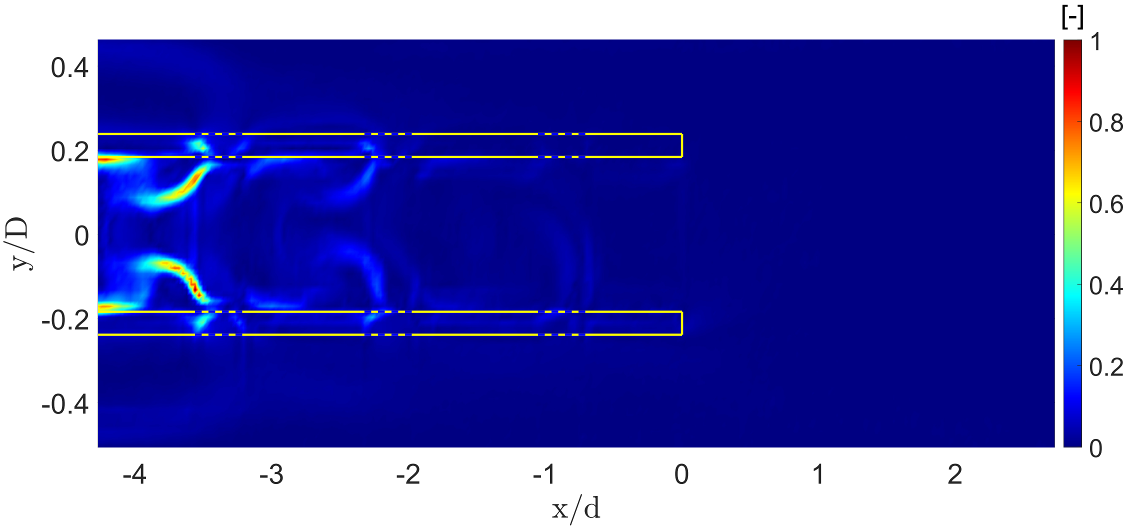

Figure 4: $Q_c = 2.6\,\mathrm{L/min}$ and $Q_o = 2.6\,\mathrm{L/min}$ ($Q = 1$)
